# Developmental variation and the evolution of distyly in *Hedyotis caerulea* (Rubiaceae)

**DOI:** 10.1186/2193-1801-2-383

**Published:** 2013-08-14

**Authors:** Dennis A Sampson, Robert A Krebs

**Affiliations:** Department of Biological, Geological, and Environmental Sciences, Cleveland State University, 2121 Euclid Ave, Cleveland, OH 44115-2406 USA

**Keywords:** Bluets, Flowers, Herkogamy, Heterostyly, *Houstonia*, Isoplethy, Floral morphology

## Abstract

The development of distyly is thought to arise from differential growth patterns in the pin and thrum morphs. However, few detailed studies exist on the early floral development of distylous flowers, and fewer still look at variation in these traits among populations. Buds at multiple stages of development were collected from five populations of *Hedyotis caerulea* to quantify how pins and thrums diverge with respect to the initiation, rate, and termination of growth between the stamens and stigmas. The growth rate of anthers varied little spatially across five populations and temporally in both pins and thrums, although thrum anthers grew faster than pin anthers. Dimorphy in stigma height was more complex. Pin stigmas first grew at a faster rate than those of thrums, and late in bud development, growth of thrum styles slowed. These rate changes varied among populations, and they differed from the congeneric *H. salzmanii*. Similar differences between morphs are known in other heterostylous species, and such variation in growth pattern among related species has been used to infer independent evolution of distylous systems.

## Introduction

Morphological variation in flowers is determined by the combined effects of genotype, developmental programming, and environmental variation (Diggle [1992]). Not surprisingly then studies of floral development have made significant contributions to understanding the evolution of plant mating systems. When available, phylogenetic hypotheses concerning the taxa studied can be merged with variation in their developmental patterns to identify possible evolutionary pathways by which new morphologies emerge (e.g., Guerrant [1982]; Hufford [1995]; Kellogg [1990]; Friedman and Carmichael [1998]; Cohen [2011]). With respect to herkogamy and one stably polymorphic form, distyly, understanding evolutionary change has been hampered by a need to track developmental change in floral development (Cohen et al. [2012]).

Fewer than 20 papers during the last 100 years have critically examined how the different forms of flowers arise in heterostylous species according to Cohen ([2010]), who noted that the majority of them focus on tristylous species. In one review, Richards and Barrett ([1992]) concluded that variation in floral organ development among families derives from independent evolutionary events that led to tristyly. While they predicted that the same could be true for distyly, they lacked information on distylous floral development in a sufficient number of species largely limited to members of the Boraginaceae (Cohen et al., [2012]), Linaceae (Armbruster et al. [2006]), Primulaceae (Stirling [1932]; Webster and Gilmartin [2006]), Santalaceae (Riveros et al. [1987]), and finally the Rubiaceae, which may be the best studied family based on reports for *Faramea suerrensis* (Richards and Barrett [1992]), *Guettarda scabra* (Richards and Koptur [1993]), *Hedyotis salzmannii* (Riveros et al. [1995]), *Psyochotria* spp and *Bouvardia ternifolia* (Faivre [2000]), and *Palicourea padifolia* (Hernandez and Ornelas [2007]).

A limited number of distylous species have been studied given that distyly occurs in some 28 different families (Barrett [2002]). At least four distinct developmental pathways have been characterized for achieving differences in stigma height between pin (long-styled) and thrum (short-styled) flowers, and at least two developmental patterns contribute to differences in anther height between the morphs (Faivre [2000]; Cohen [2010]). It is therefore likely that additional developmental pathways will be identified, and such variation among species as well as divergence among populations may help to establish what evolutionary patterns most likely underlie the origins of this trait.

Here we quantitatively compared the initiation, rate, and termination of growth between the stamens and stigmas in another member of the Rubiaceae, the distylous *Hedyotis caerulea*, and make these measurements across multiple populations. The goal was to establish developmentally when and how the floral morphology of pins and thrums diverged, to compare development to a congener and other distylous taxa, and to produce inferences on the mechanisms by which distyly evolved.

## Materials and methods

We collected 212 pin buds and 219 thrum buds at various stages of development from five populations of *H. caerulea* in northeastern Ohio in May 2008: #2, 4, 9, 10 and 13 from Sampson and Krebs ([2012]), which will be identified here as A-E, respectively. Buds were chosen in a manner that included the widest range of development possible, although the smallest buds were selected first in order to ensure their adequate representation in the data set given that *H. caerulea* possess a short growing season (Ornduff [1980]). Tiny buds were chosen from plants that already had a mature flower, which is necessary to score the individual as a pin or a thrum. All buds were preserved when fresh cut in 80% ethanol. Buds were dissected to measure bud length, stigma height, and anther height using an Olympus SZX12 dissecting microscope equipped with an ocular micrometer accurate to <0.01 mm at 90×, its highest resolution.

Bud length was examined as a relative estimate of developmental time to which stigma height and anther height were compared in pin and thrum morphs. Bud size was the independent variable and floral organ measurements were the dependent variables, and both were log-transformed prior to regression analyses to normalize data under the assumption that variance correlates with the mean. Raw measurements, however, were also presented for clarity and contrast. Size of developing stigmas and anthers were contrasted against total bud length using both a linear and a second-order term in models to test whether the relationship could best be described as curvilinear or linear, and to improve the power of this particular test, results of all five populations were pooled. Relationships among populations were tested in general linear models (Proc GLM; SAS Institute [2002]) to assess variation among populations as fixed factors and to test interactions between morphs and populations on both anther and stigma height.

## Results

**Figure 1:**
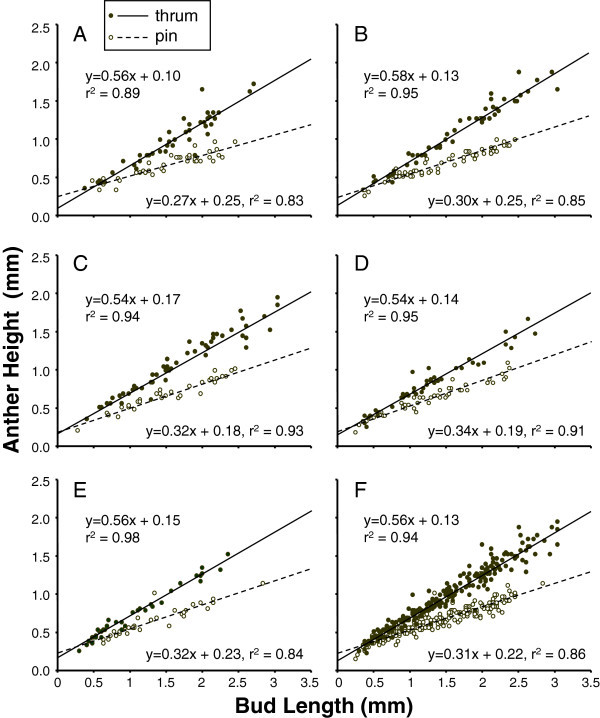
**Anther development in pin and thrum floral morphs of*H. caerulea.*** Data of bud lengths (mm) versus height of anthers (mm) were plotted for each bud collected across five populations **(A**-**E)**, and **F** provides results for all populations combined, and lines of best fit are simple linear regression of bud length predicting anther height.

**Figure 2:**
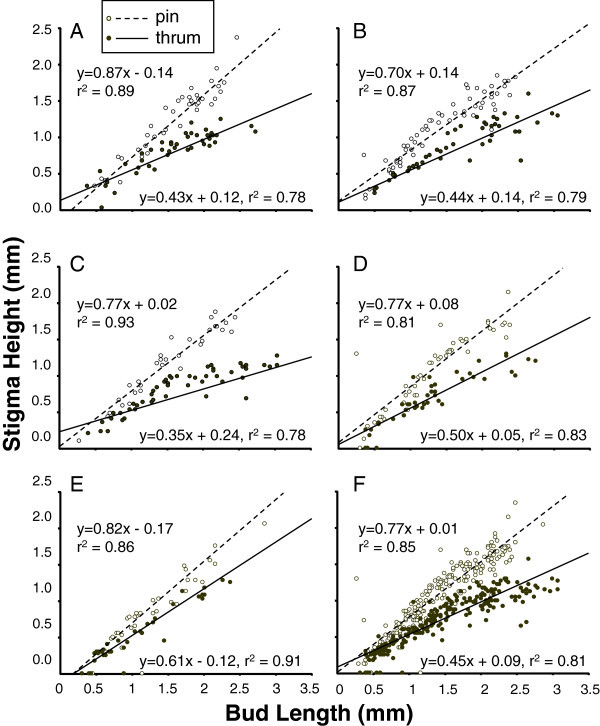
**Stigma development in pin and thrum floral morphs of*H. caerulea.*** Data of bud lengths (mm) versus height of the stigmas (mm) were plotted for each bud collected across five populations **(A**-**E)**, and **F** provides results for all populations combined, and lines of best fit are simple linear regression of bud length predicting stigma height.

Variation in growth rate between pins and thrums occurred very early in development (Figures 1 and 2). Across the five populations sampled, the linear term explained most of the variation in the relationship between bud length and anther height in both thrum and pin flowers (Figure 1, linear regressions on raw data are provided in the figure). Results were similar where data were log transformed, and for all five populations combined, a step that provided the power necessary to test for a fit of a second-order term on anther height, no improvement in the model was observed for either morph (Table 1). Thus, the growth of anther height relative to bud length was described by a best fit linear equation where the slope differed significantly between morphs (F_1,411_ = 21.6, P < 0.001), but minimally among populations. Slopes of both morphs also were significantly (P < 0.001) less than one. In both morphs, the filament served only to attach the anther to the inner surface of the corolla tube and made no contribution to the height of the anther.

**Table 1:** **Linear and curvilinear regression analysis of relative growth rate (X) of anther heights and stigma heights against bud length, for pin and thrum floral morphs**

A. Linear regression						
**Anthers**	**X**		**y-intercept**	**P(x)**	**r**^**2**^	**N**
**Pin**	0.43 ± 0.01		0.13 ± 0.01	<0.0001	0.88	212
**Thrum**	0.69 ± 0.01		0.05 ± 0.01	<0.0001	0.95	219
**Stigmas**						
**Pin**	0.91 ± 0.03		−0.06 ± 0.02	<0.0001	0.84	212
**Thrum**	0.67 ± 0.02		−0.04 ± 0.02	<0.0001	0.84	219
**B. Polynomial regression**						
**Anthers**	***X***^***2***^	***X***	***y-intercept***	***P(x***^***2***^***)***	***P(x)***	***r***^***2***^
**Pin**	−0.01 ± 0.04	0.45 ± 0.07	0.12 ± 0.03	0.840	<0.0001	0.88
**Thrum**	0.09 ± 0.04	0.54 ± 0.06	0.11 ± 0.03	0.019	<0.0001	0.95
**Stigmas**						
**Pin**	−0.04 ± 0.10	0.97 ± 0.17	−0.08 ± 0.06	0.706	<0.0001	0.84
**Thrum**	−0.43 ± 0.06	1.40 ± 0.11	−0.31 ± 0.04	<0.0001	<0.0001	0.87

Regression equations are based on log transformed data combined for the five populations of *H. caerulea*.

Intermorph differences in stigma development were less straightforward than those for anthers, although for all populations a regression of bud length of stigma height explained at least 78% of the variance (Figure 2, linear regressions on raw data are provided in the figure). To test for the fit of a second-order term, log-transformed results were again combined for all five populations. In pin flowers, the slope based on linear regression for bud length on stigma height explained 84% of the variation, and no improvement occurred by adding a second-order term (Table 1). In thrum flowers, the slope from linear regression similarly also explained 84% of the variance in thrum flowers, but here a second-order term was significant, and it improved the predictive fit of growth patterns to 87% of the variance (Table 1B).

Thus, for thrum flowers, shape and magnitude of growth rate of stigmas suggested curvilinear characteristics, which could be partitioned to examine early growth (bud lengths up to 1.5 mm) and later growth (regressing bud length on stigma height only for buds greater than 1.5 mm), using log transformed data. Basically, growth began at a rate significantly faster than one (2.44 ± 0.30, t_116_ = 4.86, P < 0.001, for the range 0–1.5 mm) and then tapered off to 0.38 ± 0.08, and while still significantly above zero (t_99_ = 5.0, P < 0.001), a temporal shift in growth rate based on differences in slope was significant (t_132_ = 6.6, P < 0.001, under assumptions of unequal sample sizes and variances).

Among the five populations, growth in anther and stigma heights followed similar trends (Figures 1 and 2). Neither population as a main effect nor the interaction between population and bud length was significant for anther height. Population variation in stigma height, however, was significant both as a main effect (F_4,411_ = 6.4, P < 0.001) and as an interaction with bud length (F_4,411_ = 4.1, P < 0.01), indicating that variation in slopes occurred among populations. However, no interactions between population and floral morph were detected, and the amount of variation explained by differences in the slope between pins and thrums remained several times greater than variation attributable to population [sum-of-squares for differences in the bud length on stigma height slope between morphs was 0.42 (df = 1), while population variation in slope was just 0.12 (with df = 4)].

## Discussion

Growth rate was uniform in anthers for both pins and thrums, but as expected, thrum anthers grew faster than pin anthers. Thus, dimorphy in anther height developed from continuous variation between morphs in growth rates beginning in the early stages of bud elongation. Species with similar anther developmental variation to that of *H. caerulea* include its congener, *H. salzmanii* (Riveros et al. [1995]) as well as *Guettarda scabra* (Rubiaceae) (Richards and Koptur [1993]), *Psychotria chiapenis*, *P. poeppigiana* and *Bouvardia ternifolia* (Rubiaceae) (Faivre [2000]), *Primula vulgaris* (Webster and Gilmartin [2006]), *Quinchamalium chilense* (Santalaceae) (Riveros et al. [1987]), and species of *Lithospermum* (Cohen [2011]). The only reported additional source of variation in anther height derives from differences in growth of filaments, where anthers are attached to the corolla. Faivre ([2000]) observed slightly longer filaments in the short-styled (thrum) forms of *P. chiapenis* and *P. poeppigiana*, but specifically not in *B. ternifolia*. Thus both growth patterns for anthers are found within the family Rubiaceae, although in *H. caerulea*, filament length did not affect anther height. Riveros et al. ([1995]). did not report on this trait in *H. salzmanii* Faivre ([2000]) suggests that measurement of filaments is easier in larger flowers perhaps explaining its limited use.

The two distylous *Hedyotis* species, however, differ in their patterns of stigma development. *Hedyotis caerulea* follows perhaps the most common model, as described for *Lithospermum* in the Boraginaneae (see Figure three in Cohen et al. [2012]): in thrums, as the bud continues to grow, elongation of the stigma slows down or possibly levels off, producing a curvilinear growth pattern described by a second-order equation; in pins the stigma elongates at a constant (linear) rate. The complex stigma development of *H. caerulea* is not unique in the Rubiaceae, as a similar pattern was observed in *Guettarda scabra* (Richards and Koptur [1993]). But, in *H. salzmannii* (Riveros et al. [1995]) as well as in *P. chiapenis*, *P. poeppigiana* and *B*. *ternifolia* (Faivre [2000]), differences in stigma height between morphs appear to arise from uniform variation in stylar growth.

The presence of different growth mechanisms has been applied to infer independent origins of heterostyly (Barrett [2002]), and here would suggest separate evolution of distyly in *H. caerulea* and *H. salzmanii*. The question is how many variations on one theme can come about and why variation can be extensive even within a species (Sosenski et al. [2010]). The answer may be changes based on existing variation, which, as Cohen et al. ([2012]) suggest, alleviate dependence on models requiring new mutations to produce herkogamy (e.g., Charlesworth and Charlesworth [1979]; Barrett [1992]; Lloyd and Webb [1992]). They advocate a model modification described by Sakai and Toquenaga ([2004]), in that each breeding system originates from the genetic variation available in traits underlying pollination success. As an example. Baena-Diaz et al. ([2012]) suggest that diversity in stamen and style heights among populations of *Oxalis alpine* (Oxalidaceae), which are now exposed to limited pollinators, will be pushed by selection from the tristylous condition towards distyly. Keller et al. ([2012]) also highlight the potential importance of population level variation similar to what we found in *H. caerulea* (here and in Sampson and Krebs [2012]) to provide the basis for shifts towards distyly. Presently, population variation in distylous species is understudied, but occurs in some Boraginaceae (Ferrero et al. [2011]) and the Iridaceae (Sánchez et al. [2010]), but little was made of its presence. Clearly, more information on genetic variation and especially genetic linkage of traits related to distyly would be useful for discriminating among existing models (e.g., Darwin [1877]; Lloyd and Webb [1992]; Sakai and Toquenaga [2004]) for the evolution of these complex mating systems.
